# Housing conditions and *Plasmodium falciparum *infection: protective effect of iron-sheet roofed houses

**DOI:** 10.1186/1475-2875-5-8

**Published:** 2006-02-01

**Authors:** Yazoumé Yé, Moshe Hoshen, Valérie Louis, Simboro Séraphin, Issouf Traoré, Rainer Sauerborn

**Affiliations:** 1 Nouna Health Research Center (CRSN), P.O. Box 02, Nouna, Burkina Faso, Germany; 2 School of Public Health and Community Medicine, Braun Hebrew University-Hadassah, Jerusalem, Israel; 3 Department of Tropical Hygiene and Public Health, University of Heidelberg-Medical School, Im Neuenheimer Feld 324, D-69120 Heidelberg, Germany

## Abstract

**Background:**

Identification and better understanding of potential risk factors for malaria are important for targeted and cost-effective health interventions. Housing conditions have been suggested as one of the potential risk factors. This study aims to further investigate this risk factor, and is focused on the effect of the type of roof on *Plasmodium falciparum *infection among children below five years in the North West of Burkina Faso.

**Methods:**

In a cross-sectional study design, 661 children aged six to 60 months were randomly selected from three rural and one semi-urban site at the end of the rainy season (November 2003). The children were screened for fever and tested for *Plasmodium falciparum *infection. In addition, data on bed net use and house characteristics was collected from the household were each child lived. Using adjusted odds ratios, children living in house roofed with iron-sheet were compared with those in house with mud or grass roof.

**Results:**

Overall *P. falciparum *infection prevalence was 22.8 % with a significant variation between (Chi-square, p < 0.0001). The prevalence in Cissé (33.3 %) and Goni (30.6 %) were twice times more than in Nouna (15.2 %) and Kodougou (13.2 %). After adjusting for age, sex, use of bed net and housing conditions, children living in houses with mud roofs had significantly higher risk of getting *P. falciparum *infection compared to those living in iron-sheet roofed houses (Odds Ratio 2.6; 95% Confidence Interval, 1.4–4.7).

**Conclusion:**

These results suggest that house characteristics should be taken into consideration when designing health intervention against *P. falciparum *infection and particular attention should be paid to children living in houses with mud roofs.

## Background

Malaria, a preventable disease, is still one of the most important causes of morbidity and mortality in developing countries, especially in sub Saharan Africa [1]. For decades, a number of initiatives have been undertaken to control the disease and reduce it related burden. Unfortunately, the success of these actions remains restricted to some specific areas [2,3]. An increase in the malaria burden is envisaged due to drug resistance, breakdown of health care systems and environmental changes [4]. The limited success of a global approach to malaria eradication can be attributed to the difference in epidemiology, environment and socio-economic conditions from one setting to another. There is, therefore, a need to understand context-specific potential risks factors associated with *Plasmodium falciparum *infection.

Among the list of risk factors, ranging from biological, physical environment, health care systems and socio-economic conditions, the design of a house significantly affects the incidence *P. falciparum *infection [5-8]. Gamage-Mendis *et al *[5] demonstrated that in Sri Lanka, living in a completed house with brick and plaster walls and tiled roofs was highly associated with malaria risk reduction compared to living in the poorest type of house. In a randomized controlled trial in rural Gambian, Lindsay *et al *[7] found that adding ceilings in mud huts reduced the number of *Anopheles *mosquitoes and other vectors entering the room and may be an effective way to reduce malaria risk.

Better housing is one of the factors that reduced malaria infection risk in regions that used to be endemic and modification of houses was used to protect against malaria in Italy, Greece, Panama and the USA in the early 20th century [9]. Alongside life style and local habits, housing conditions play a role in modulating exposure of populations to mosquitoes and vector-borne diseases [10].

Roof types determine indoor temperature, a parameter that is associated with malaria transmission. The objective of this study was to investigate the effect of the type of roof on *P. falciparum *among children below five years of age in the North West of Burkina Faso. This was done because, considering current knowledge, no study has specifically assessed and quantified the effect of different types of roofs upon *P. falciparum *infection in a sub-Saharan setting.

## Materials and methods

### Study area

**Figure 1:**
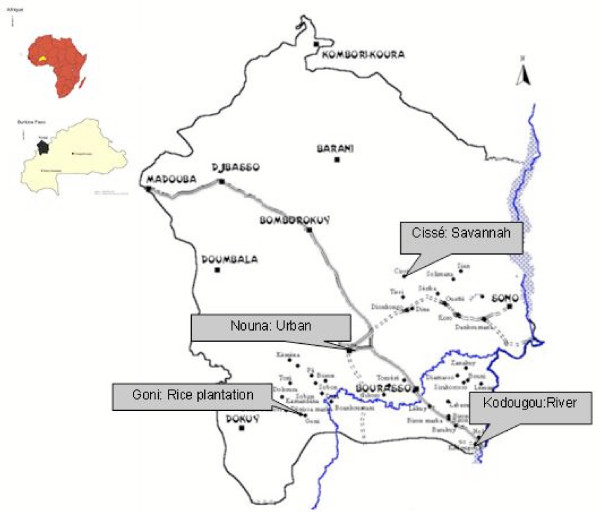
Location of the study sites in Nouna Health district (Kossi province).

The study area has dry savannah vegetation, with a hot and short rainy season. The total annual rainfall is about 700 mm concentrated in the June-September period. The average annual temperature is 29°C with a wide seasonal variation and diurnal variation in the cold period (December-January). In November, the mean temperature is around 29°C with a diurnal variation of 21°C to 38°C. Rainfall is almost inexistent, but some residual breeding sites from the rainy season remain and are sources of mosquitoes. Due to the climate conditions, malaria transmission, which is holoendemic in this area, has a strong seasonal variation with a high transmission period that starts one month after the onset of the rainy season until November [11]. Four study sites representing different ecological settings were selected (Figure 1). Nouna was a semi-urban setting. Cissé, Kodougou and Goni were rural settings located by a protected savannah forest, a perennial river and a rice-farming plain respectively.

All the sites were part of a Demographic Surveillance System (DSS), which covers the Nouna health district located in the province of Kossi. In this area, since 1992 the population is followed longitudinally for demographical events (birth, death, in and out-migration) [12]. These data were made available for this study.

**Figure 2:**
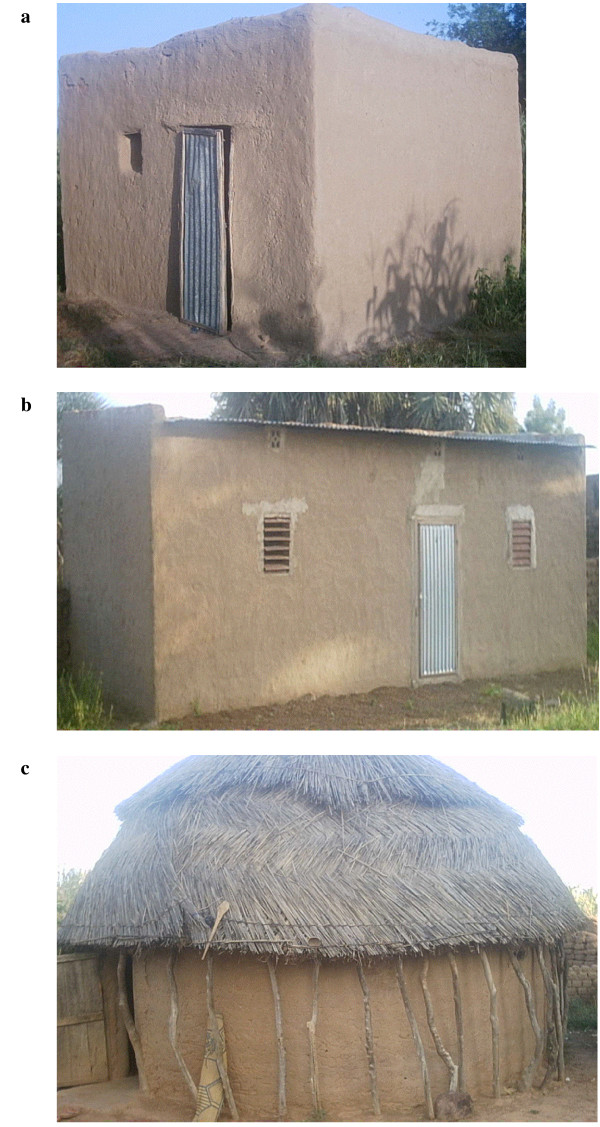
Typical housing in study region; A) mud blocks with mud roof; B) mud blocks with iron sheet roof; C) mud blocks with grass roof.

Houses are mainly constructed out of local materials. About 80% of the houses were built of mud blocks and roofed with the same material (Figure 2a). Some mud block houses were roofed with iron-sheets as well as grass (Figure 2b and 2c). There were more houses with iron-sheets in the semi-urban settings than in the rural ones.

### Study population

Study participants (661) were children aged between six and 60 months from the four sites. They were selected randomly through cluster sampling of households (352), from the four sites, the sampling frame of which was obtained from the Nouna DSS database.

Parents of selected children were visited prior to the survey to obtain informed consent. The objective and methods of the study were explained to them during these visits. They were also told that the finger prick procedure was very safe with the use of single-use lancets, although it may be slightly painful for the children; that the quantity of blood taken with this method was not harmful for the child; that the child would benefit from a medical check-up for malaria and other common pathology; that medical treatment would be offered free of charge if needed; and that referral was possible for severe cases.

### Detection of infection

Malaria infection data were collected by active case detection from each of the four sites from the 14^th ^to 18^th ^November 2003. A mobile team of one physician, one nurse, two laboratory technicians and five interviewers visited each site. Mothers were asked to bring their children to a meeting point identified by the village chief. Children were screened for fever using digital axillary thermometers. Febrile (body temperature > = 37.5°C) children were physically examined, including weight measurement and spleen palpation and then treated with chloroquine (25 mg/kg weight during three days: day one: 10 mg/kg, single intake; day two: 10 mg/kg, single intake; day three: 5 mg/kg) and paracetamol. A blood sample was then taken using the finger prick method. Severely sick children were referred to the nearest health unit. The slides were later stained with Giemsa and read for parasite count in the laboratory of the "Centre de Recherche en Santé de Nouna" (CRSN). Parasite density was estimated by counting 100 fields and equating this to 0.25 μl of blood [13]. Each child with any *P. falciparum *detected was classified as infected.

In addition, mothers were asked if the child had been sleeping under a mosquito net, and whether the mosquito net was treated or not with insecticide. Use of anti-malarial drugs in the previous week was also noted.

### Housing conditions data

After the completion of the clinical phase, mothers were visited at home by the interviewers for detailed interviews about their housing conditions. Since a typical household compound in this area is comprised of several houses, the house where the participating child usually slept was selected. Using a structured questionnaire, the interviewer asked about the type of material used for the wall (mud block, grass, stone, or cement bricks) and the roof (iron-sheet, mud or grass). They also asked about the presence of animals, well and potential mosquito breeding site (open water body) within 50-meter radius of the house. All answers were cross-checked by observation.

### Data management and statistical analysis

Proc logistic procedure was used to estimate the odds ratio (OR) of living in a house with a mud roof and one with a grass roof compared to living in a house with iron-sheets. The multivariate model included potential confounders such as age group (categorical: <12, 12–23, 24–60 months), location of the house (categorical: Cissé, Goni, Kodougou, or Nouna), presence of animal (binary: yes or no), presence of a well (binary: yes or no), presence of breeding sites and use of mosquito net (binary Yes or No). The logistic regression model was defined as follows:

log*it*(*π*_*i*_) = *β*_0 _+ *β*_1_*Roof_earth*_*i *_+ *β*_2 _*Roof_grass*_*i *_+ *β*_3 _*X*_*i *_+ *β*_4 _*X*_*i *_+...+ *β*_*x *_*X*_*i *_

Where *π*_*i *_is the predicted probability for the *i*th child, of being positive *Pf *infection or having clinical malaria. The odds of the same child will be , *β*_0 _the intercept, and *β*_1 _... *β*_*x *_the regression coefficients of the independent variables (name following each coefficient *X*_*i *_being the potential confounders mentioned above). The odds ratio associated with *Roof_earth *compared to iron-sheet roof (reference) is the exponential of *β*_1 _(OR_*Roof_earth *_= exp(*β*_1 _)). The significance of the OR was assessed using 95% confidence intervals (CI). All statistical analysis was done using SAS version 9.1 (Cary, NC USA).

## Results

### Population characteristics

The descriptive characteristics of the study population are given in Table 1. The average number of children enrolled in the study, per household, was 1.9. Sex and age distribution in the four sites were similar (Chi-square, p = 0.1703, p = 0.5738 respectively) Overall, females were slightly overrepresented with 51.8% but the difference was not statistically significant (Chi-square, p = 0.3710). The majority of the children were within the age group of 24–60 months (68.9%), 22.4% were aged between 12 and 23 months and 8.8 % were below 12 months. Children were distributed in five ethnic groups: Fulani, Bwaba, Mossi, Samo and Marka. Overall the Marka (30.8%), Mossi (29.1%) and Fulani (21.8%) were predominant. Except in Nouna where all the five ethnic groups were well represented, each village was characterized by one dominant ethnic group (Fulani: 83.7% in Cissé, Marka: 69.7% in Goni and Mossi: 72.2% for Kodougou).

**Table 1:** Distribution of the study participants, by site, sex and age, November 2003

	**Sites**				
	
	**Cissé (%)**	**Goni (%)**	**Kodougou (%)**	**Nouna (%)**	**Total (%)**
**N**	**141**	**183**	**151**	**186**	**661**
**# of Households**	67	107	64	114	352
**Sex**					
Female	82 (58.2)	84 (46)	77 (51.0)	99 (53.3)	342 (51.8)
Male	59 (41.9)	99 (54.1)	74 (49.1)	87 (46.8)	319 (48.3)
**Age (months)**					
<12	11 (7.9)	15 (8.2)	10 (6.7)	22 (11.9)	58 (8.8)
12–23	28 (19.9)	45 (24.6)	37 (24.6)	38 (20.5)	148 (22.4)
24–60	102 (72.4)	123 (67.3)	104 (68.9)	126 (67.8)	455 (68.9)
Median	34	32	34	31	32.6
**Ethnic groups**					
Fulani	118 (83.7)	5 (2.8)	0 (0)	21 (11.3)	144 (21.8)
Bwaba	2 (1.5)	4 (2.2)	27 (17.9)	21 (11.3)	54 (8.2)
Mossi	0 (0)	47 (25.7)	109 (72.2)	36 (19.4)	192 (29.1)
Samo	4 (2.9)	0 (0)	14 (9.3)	41 (22.1)	59 (9)
Marka	14 (10)	127 (69.4)	1 (0.7)	61 (32.8)	203 (30.8)
Other	2 (1.5)	0 (0)	0 (0)	6 (3.3)	8 (1.3)

### Housing conditions

A majority (75.3 %) of children lived in mud roofed houses. The same distribution was observed in all the four sites (Table 2). In contrast, there was large variation for iron-sheet roofed houses with Nouna (36.6 %) and Goni (30.1 %) having the highest proportion, while Cissé has only 2.2 % and Kodougou none. Overall there were few (6.6 %) children sleeping under grass roofed houses.

**Table 2:** Distribution of participants according to bed net use and housing conditions, November 2003

	**Sites**				
	
	**Cissé (%)**	**Goni (%)**	**Kodougou (%)**	**Nouna (%)**	**Total (%)**
**N**	**141**	**183**	**151**	**186**	**661**
**Roof type**					
Iron-sheet	3 (2.2)	55 (30.1)	0 (0)	68 (36.6)	126 (19.1)
Mud	128 (90.8)	124 (67.8)	133 (88.1)	113 (60.8)	498 (75.3)
Grass	10 (7.1)	4 (2.2)	18 (12)	5 (2.7)	37 (5.6)
**Reported use of bed net**					
Yes	73 (51.8)	147 (80.4)	103 (68.3)	29 (15.6)	352 (53.3)
No	65 (46.1)	34 (18.6)	44 (29.2)	141 (75.9)	284 (43)
Missing	3 (2.2)	2 (1.1)	4 (2.7)	16 (8.7)	25 (3.8)
**Animal within 50 m radius of the house**					
Yes	114 (80.9)	123 (67.3)	50 (33.2)	122 (65.6)	409 (61.9)
No	27 (19.2)	60 (32.8)	101 (66.9)	64 (34.5)	252 (38.1)
**Well within 50 m radius of the house**					
Yes	28 (19.9)	0 (0)	0 (0)	94 (50.6)	122 (18.5)
No	113 (80.2)	183 (100)	151 (100)	92 (49.5)	539 (81.5)
**Breeding site within 50 m radius of the house**					
Yes	0 (0)	13 (7.2)	0 (0)	76 (40.9)	89 (13.5)
No	141 (100)	170 (92.9)	151 (100)	110 (59.2)	572 (86.5)

Reported mosquitoes net use among participants was relatively high (53.3 %) but varied significantly (chi-square, p < 0.0001) between sites with the highest coverage in Goni (80.4 %) and Kodougou (68.3 %). Nouna had surprisingly the lowest coverage (15.6 %). Data on mosquito net use was missing for 3.8 % of the children and these were eliminated from subsequent analyses.

Overall, 61.9 % of children lived in a house with an animal enclosure within a 50-meter radius but there was significant variation between the sites with a maximum in Cissé of 80.9 % (Chi-square, p < 0.0001). 18.5 % of households had wells and 13.5 % had a potential breeding site within a 50-meter radius of the house. In Nouna these figures were significantly higher than at the others sites 50.6 % and 40.9 % respectively (Table 2).

### *P. falciparum *infection

The distribution of *P. falciparum *infection among participants is given in Table 3. The overall *P. falciparum *infection prevalence was 22.8 % with a significant difference (Chi-square, p < 0.0001) between the sites. The prevalence was more than two times higher in Cissé (33.3 %) and Goni (30.6 %) compared to Kodougou (13.4 %) and Nouna (15.1 %). There was no significant difference of *P. falciparum *infection between males (21.9 %) and females (23.8 %). Children aged between 12 and 23 months experienced the highest prevalence of *Plasmodium falciparum *(27.0 %) compared to those below 12 months (17.2 %) and above 23 months (14.3 %). The *P. falciparum *prevalence was high among children living in mud roofed houses, overall (25.9%) and consistently at all sites.

**Table 3:** Distribution of *P. falciparum *infection prevalence among study participants by sex, age and site, November 2003

	**Sites**				
	
	**Cissé (%)**	**Goni (%)**	**Kodougou (%)**	**Nouna (%)**	**Total (%)**
**n**	47 (33.3)	56 (30.6)	20 (13.3)	28 (15.1)	151 (22.8)
**Sex**					
Female	19 (23.7)	29 (34.5)	12 (15.6)	15 (15.1)	75 (21.9)
Male	28 (47.5)	27 (27.3)	8 (10.8)	13 (14.9)	76 (23.8)
**Age (month)**					
<12	3 (27.3)	4 (26.7)	0 (0.0)	3 (13.6)	10 (17.2)
12–23	11 (39.3)	16 (35.6)	9 (24.3)	4 (10.5)	40 (27.0)
24–60	33 (32.4)	36 (29.3)	11 (10.6)	21 (16.7)	101 (22.2)
**Roof type**					
Iron-sheet	0 (0)	9 (16.4)	0 (0.0)	7 (10.3)	16 (12.7)
Mud	44 (34.4)	46 (37.1)	20 (15.0)	19 (16.8)	129 (25.9)
Grass	3 (30.0)	1 (25.1)	0 (0.0)	2 (40.0)	6 (16.2)

### Effect of roof type on *P. falciparum *infection risk

The prevalence of *P. falciparum *infection among participants who lived in iron-sheet roofed houses (12.7 %) was two times less than those who lived in mud roofed houses (25.6 %). A logistic regression model, controlling for site of residence, mosquito net use, presence of well, animal enclosure and potential mosquitoes breeding sites showed that inhabitants of houses with mud roof had significantly higher risk (OR 2.6, 95% CI 1.4–4.7) of getting *P. falciparum *infection compared to those living in iron-sheet roofed houses (Table 4). An intermediate prevalence (16.2 %) was observed among children living in grass roofed houses. In this case, the OR for *P. falciparum *infection was high (1.7) but not statistically significant.

**Table 4:** Effect of house roof on *Plasmodium falciparum *infection among children

				**Crude**		**Adjusted	
				
**Roof**	**n**	**cases**	**Prevalence**	**Odds Ratio**	**95%CI**	**Odds Ratio**	**95%CI**
				
*Iron-sheet	126	16	12.7	1		1	
Mud	498	129	25.9	**2.4**	(1.3–4.2)	**2.6**	(1.4–4.7)
Grass	37	6	16.2	1.3	(0.4–3.6)	1.7	(0.5–5.0)
Total	661	151	22.8				

* Reference category, ** Controlled for site, bednet use and presence of breeding site, well, animal enclosure

## Discussion

One of the principal issues in public health is the question of equity of treatment and variation in vulnerability of the relevant population. Of special relevance is the effect of socioeconomic class on the risk of morbidity and mortality. Malaria as a "poor man's disease", which affects either the children or parents in poverty stricken households of sub-Saharan Africa, epitomizes the problem. In this research one characteristic of financial status was tested, the quality of housing. Corrugated iron-sheets are relatively expensive and cannot be afforded by most villagers, in spite of their better protection from dampness. The question was whether this roofing material would also protect against malaria. There were two conflicting hypotheses as to the influence of the roof on the preponderance of house-dwelling mosquitoes, and hence on mosquito prevalence: (a) the iron-sheet would increase prevalence, due to the heating of the roof and resulting in increased indoor temperature and (b) the iron would decrease prevalence as the roof would no longer be a suitable resting place for blood-engorged mosquito, as opposed to cracks in the mud roof, and this would disturb the malaria-transmission cycle.

The finding that there is a significant increase in malaria risk in mud roofed huts, after adjusting for other environmental conditions, suggests that the poorer sections of the population are at greater risk and thus require more intensive prevention. In this case it seems that internal spraying of roof areas would be useful. In addition, in areas where these types of dwellings are common, intensive surveillance would be required to reduce the high levels of child morbidity and mortality. From a biological point of view, it is expected that the microclimate conditions in grass roofed houses are more or less similar to the ones in mud roofed houses and similar precautions should be recommended. The non-significance of the results for the grass roofed houses in this case may be attributable to the small proportion of children living in this type of house, resulting in low statistical power.

At a more theoretical level, weather-based models of malaria incidence, prevalence and mortality, require a clear evaluation of the ambient conditions of the mosquito environment. While it has been suggested that inside-outside temperature differentials are important, the results here suggest that the structural conditions are of greater importance.

Other possible explanations for the differences in prevalence between roof structures may also be raised. For instance, the more affluent houses may have better sealing around doors, hence preventing entry of mosquitoes. In addition, in the more affluent households children may be better nourished and hence less susceptible to infection. On the other hand, socioeconomic status would not have had much impact on the results since the main outcome was *P. falciparum *infection rather clinical malaria. The latter is likely to be influenced by better access to treatment. Further research will be required to test these explanations.

## Conclusion

The paper presents the risk of *P. falciparum *infection associated with the type of roof among under five children in North West of Burkina Faso, a holoendemic area. *P. falciparum *infection prevalence is consistently high in the four sites although there are differences between sites. After controlling for potential confounders, it was shown that children living in iron-sheet roofed houses have two times less risk of getting *P. falciparum *compared to those living in houses with mud roofs. This study adds evidence to the body of knowledge regarding the effect of house type on *P. falciparum *infection risk. In conclusion, taking house characteristics into consideration in any health intervention programme against malaria will be beneficial.

## Authors' contributions

Yazoumé Yé has designed and implemented the study. He did the data analysis and wrote the manuscript. Moshe Hoshen and Valérie Louis have contributed to the data analysis and writing of the manuscript. Séraphin Simboro and Issouf Traoré have contributed to the data collection. Rainer Sauerborn Contributed to the design, implementation, analysis and writing of the manuscript.
